# Effect of denture tooth manufacturing and adhesive materials on bond strength of 3D-printed denture base

**DOI:** 10.4317/jced.63377

**Published:** 2025-12-30

**Authors:** Thanatat Saengthongpinij, Pongsakorn Apinsathanon, Basel Mahardawi, Palawat Laoharungpisit, Pheeradej Na Nan, Napapa Aimjirakul

**Affiliations:** 1 DDS. Department of Conservative Dentistry and Prosthodontics, Faculty of Dentistry, Srinakharinwirot University, Bangkok, Thailand; 2 DDS, MSc. Department of Prosthodontics, Faculty of Dentistry, Chulalongkorn University, Bangkok, Thailand; 3 DDS, MSc, PhD. Department of Oral and Maxillofacial Surgery, Faculty of Dentistry, Chulalongkorn University, Bangkok, Thailand; 4 DDS, MSc, PhD. Oral and Maxillofacial Surgery and Digital Implant Surgery Research Unit, Faculty of Dentistry, Chulalongkorn University, Bangkok, Thailand; 5 BEng, MEng, MBA, PhD. Department of General Dentistry, Faculty of Dentistry, Srinakharinwirot University, Bangkok, Thailand; 6 MEng, MBA, PhD. National Cyber Security Agency, Bangkok, Thailand; 7 DDS, PhD. Department of Conservative Dentistry and Prosthodontics, Faculty of Dentistry, Srinakharinwirot University, Bangkok, Thailand

## Abstract

**Background:**

3D printing enhances denture fabrication, but the bond between printed teeth and bases is often weaker than traditional methods. This study investigates the effect of tooth-adhesive combinations on bond strength to enhance clinical reliability in digital dentistry.

**Material and Methods:**

Forty-eight cylindrical 3D-printed denture bases were bonded with either 3D-printed (PT) or milled (MT) teeth using three bonding materials: NextDent (ND), Super-Bond (SUP), and Unifast Trad (UNI)(n=8/group). The PT-ND group served as the control. Specimens were subjected to shear bond strength testing using a universal testing machine at 0.5 mm/min, with a 4 mm shear pin, and failure modes were analyzed under a stereomicroscope.

**Results:**

Tooth type, bonding material, and their interaction significantly influenced bond strength (P&lt;0.05). MT-UNI and MT-SUP demonstrated the highest values (92.33 ± 4.90 N; 87.34 ± 2.41 N), while MT-ND had the lowest (11.72 ± 1.94 N). Among PT groups, PT-SUP performed best (45.59 ± 3.47 N), followed by PT-ND (34.12 ± 3.38 N) and PT-UNI (23.68 ± 4.14 N). High-strength groups exhibited predominantly mixed failure (MT-SUP, MT-UNI, PT-SUP), while lower-strength groups showed adhesive failure. (MT-ND, PT-UNI).

**Conclusions:**

Bond strength is influenced by both tooth material and bonding agent. From the results, Unifast Trad is optimal for milled teeth; Superbond is best suited for printed teeth. Material selection is critical for improving durability and chairside efficiency in digital prosthodontics.

## Introduction

Removable dental prostheses remain essential for rehabilitating partially or completely edentulous patients, restoring masticatory function, speech, and aesthetics. While offering a cost-effective alternative to fixed prostheses, these appliances frequently fail due to debonding between denture teeth and the acrylic base (1 , 2). This mechanical failure increases clinical workload through repeated repairs and replacements. While traditional repairs require laboratory intervention, chairside solutions using adhesive materials offer a promising alternative (3). However, the efficacy of these adhesives may vary between conventional heat-cured prostheses and modern CAD/CAM-fabricated ones, particularly 3D-printed denture bases, which exhibit different surface properties and polymerization characteristics compared to milled or conventionally processed bases (4 - 5). The bonding material can influence adhesion between denture teeth and the base (6). One of the most popular adhesion materials used nowadays is resin cement. O'Brien classified the resin cements into two categories: poly(methylacrylate) and dimethylacrylate. A representative poly(methylacrylate) cement is Super-bond®, a self-curing system composed of powder and liquid components. The liquid monomer contains 4-META (4-Methacryloxyethyltrimellitate anhydride) and tri-butylborane (TBB) as a polymerization initiator, ensuring efficient curing (7). Another common material is auto-polymerized acrylic resin (e.g., UNIFAST TRAD), widely used in studies on provisional restorations and repair techniques (8). Its quick setting time and clinical convenience make it a preferred choice. These materials differ in adhesion mechanisms, polymerization shrinkage, and substrate penetration, which may impact denture tooth bonding (5). While the retention of denture teeth to conventionally processed and milled denture bases has been well-documented, limited data are available regarding the bonding efficacy to 3D-printed denture bases, particularly when comparing different denture tooth materials and bonding agents. Studies suggest CAD/CAM-produced dentures (milled or 3D-printed) exhibit lower shear bond strength than conventional heat-polymerized ones, possibly due to manufacturing differences (9 - 12). Therefore, this study evaluates the shear bond strength and failure modes of denture teeth (made via different methods) bonded to 3D-printed bases using chairside adhesives. The null hypothesis is that neither the tooth fabrication method nor the adhesive type affects bond strength.

## Material and Methods

1. Specimen preparation Forty-eight specimens were fabricated following the modified ISO/TS 19736:2017 standard (Dentistry - Bonding test between polymer teeth and denture base materials). Sample size was determined using G*Power (v3.1.9.6; Heinrich-Heine-Universität Düsseldorf, Düsseldorf, Germany) with 95% power and 5% type I error, and an effect size of 0.6 from a pilot study. The maxillary right central incisor (Majordent®, Major Prodotti Dentari S.P.A., Via Einaudi, Moncalieri (TO), ITALY) was scanned (3Shape E4 scanner, 3Shape Inc., Holmens Kanal, Copenhagen K, Denmark) to generate an STL file for fabricating experimental teeth. Twenty-four printed teeth were produced using NextDent C&amp;B MFH resin (shade N1, Vertex-Dental B.V., Centurionbaan, Soesterberg, The Netherlands) on a NextDent 5100 3D printer (Vertex-Dental B.V., Centurionbaan, Soesterberg, The Netherlands), while another 24 were milled from a multi-PMMA disk (Upcera, shade A2, UPCERA DENTAL AMERICA INC, Alondra, Cerritos, CA, United States) using a vhf S2 milling machine (vhf camfacture AG, Lettenstraße, Ammerbuch, Germany). Cylindrical denture bases, 20 mm diameter, 20 mm height were designed by Autodesk Tinkercad 3D design software (Autodesk, Inc., San Francisco, CA, USA.) with a 1 mm recess for tooth attachment and printed using NextDent Denture 3D+ resin (Vertex-Dental B.V., Centurionbaan, Soesterberg, The Netherlands)with a 90° orientation, UV-cured. Post-processing included ultrasonic cleaning (99% isopropyl alcohol, 5 min) and UV curing for 30 min, 60°C (NextDent® LC 3DPrint Box, 3D Systems, USA). Supporting structures were removed, and non-bonding surfaces were polished. 2. Bonding of denture tooth All materials used for the union of denture tooth to denture base in this study are shown in Table 1.

Table 1

**Figure 1:**
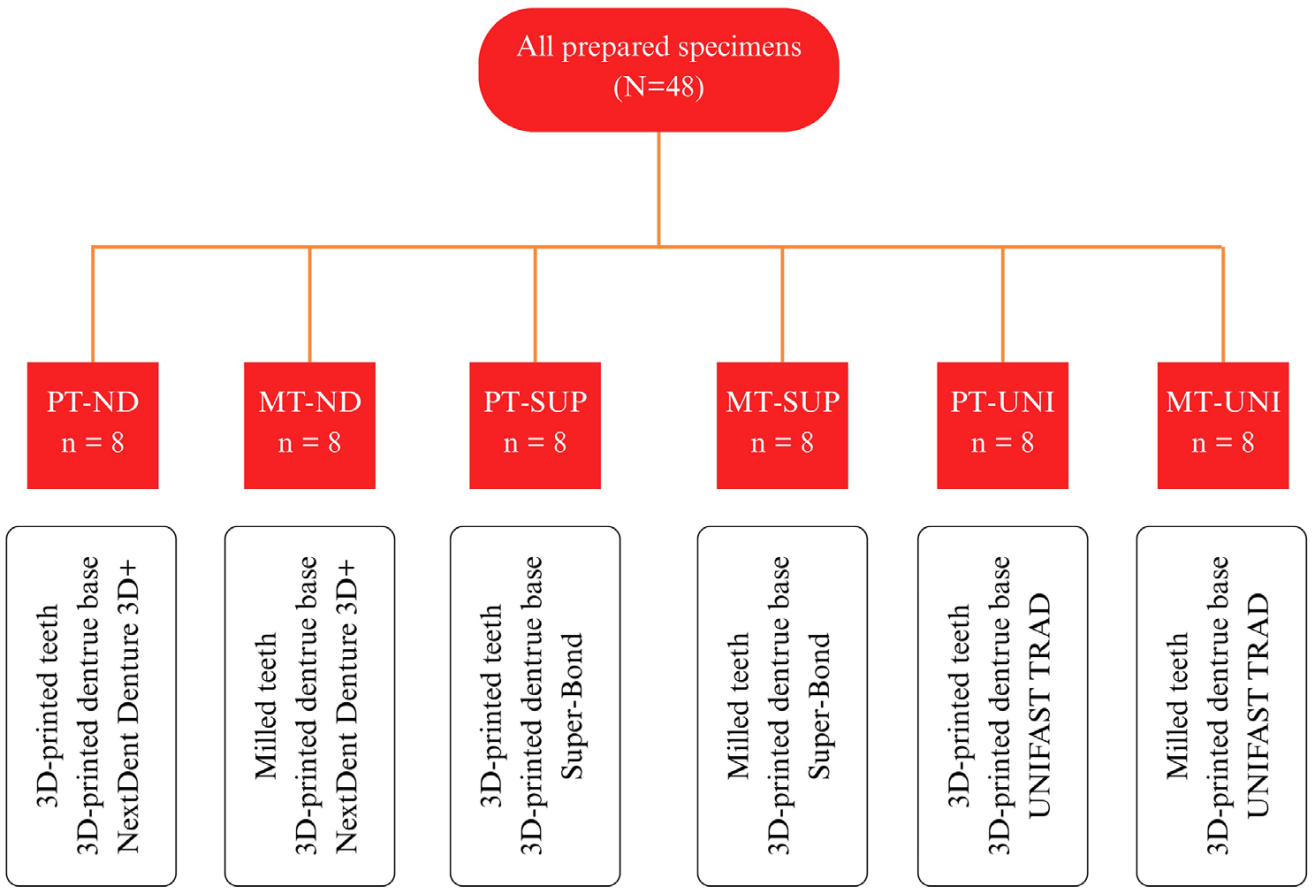
Experimental group.

Clear adhesive tape was applied to the unbonded surface to facilitate the subsequent removal of excess bonding material. Specimens were randomly assigned to bonding groups (n=8/group): printed teeth with NextDent resin (PT-ND), Super-bond C&amp;B (PT-SUP), or UNIFAST TRAD (PT-UNI), and milled teeth with the same materials (MT-ND, MT-SUP, MT-UNI) (Fig. 1).

1

**Figure 2:**
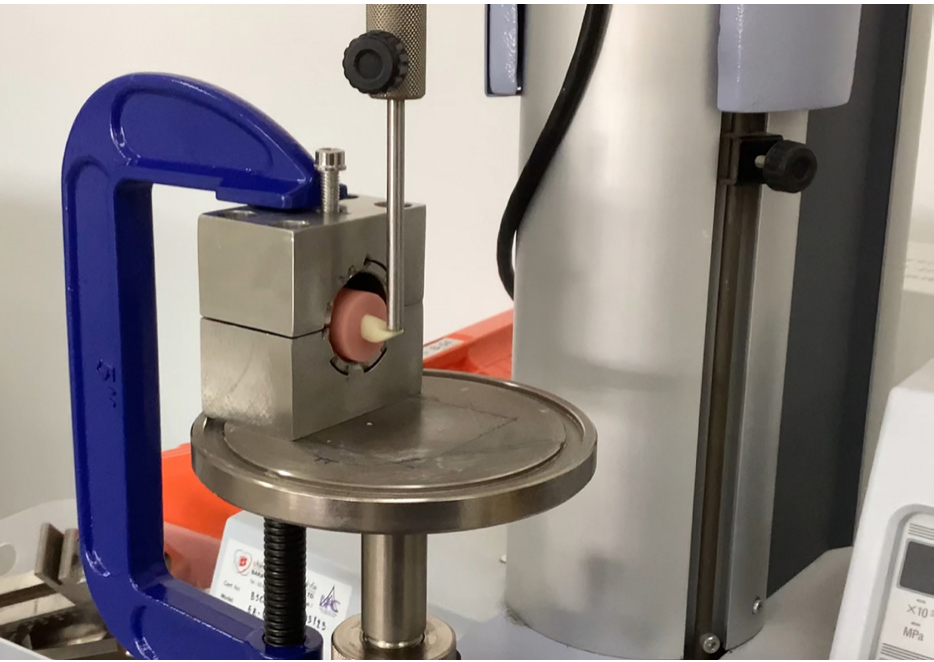
Specimen positioning for the bonding test.

Bonding protocols followed manufacturers' instructions: light-curing (NextDent Denture 3D+), chemical curing (Super-bond), or auto-polymerizing (Unifast Trad). For NextDent Denture 3D+ dental resin, all denture bases and teeth specimens were cleaned with ethanol in the ultrasonic bath for three minutes. A small amount of LC-Retention resin was dispensed onto the bonding area of the denture bases by a dropper. The artificial teeth were placed in the correct position and pressed gently to ensure proper adaptation. An excess of resin was removed by a micro-brush before curing. A light-curing unit was used to cure for 60 seconds on each side of the artificial teeth, including buccal, lingual, mesial, and distal. For using Super-bond C&amp;B as a bonding material, four drops of the monomer were mixed with one drop of the Catalyst V using a light stirring with a brush. A small cup of Super-bond's measuring spoon (0.2 mL) of the Polymer powder was then added to the liquid that had already been mixed. The mixed liquid was immediately applied to the surface to be bonded using a brush. After setting the teeth, they were held in position for five minutes. Lastly, the excess resin was wiped off with a brush soaked in alcohol. In terms of using Unifast Trad as a bonding material, a small amount of powder was added to the liquid and mixed thoroughly to avoid air bubbles until reaching a 2:1 by weight of powder: liquid ratio using equipment provided by the manufacturer. Mixing was continued until a smooth and homogenous consistency was achieved within 15 seconds. The mixed material was applied to the bonding area by brush. Finally, all specimens were stored in distilled water within a temperature-regulated cabinet (37 ± 1°C) for 24 hours before bond strength testing. 3. Bond strength test and Failure mode determination Shear bond strength (Fig. 2) was tested with a universal testing machine (Shimadzu AGS-X) at 0.5 mm/min, with a 4 mm shear pin applied 3 mm gingivally from the incisal edge, perpendicular to the lingual surface of the tooth specimens to simulate the centric occlusion contact point of Angle's Class I occlusion (13).

2

The shear force (N) at failure was recorded for each specimen. Failure modes (adhesive, cohesive, mixed) were assessed via a stereomicroscope (Olympus, SZX7 Stereomicroscope, Olympus Corporation, Hachioji, Tokyo, Japan) at 10X magnification. 4. Statistical Analysis The statistical analysis was conducted using statistical software SPSS Statistics Version 25.0(SPSS Inc., Illinois, United States). Normality of the data distribution was assessed using the Shapiro-Wilk test due to the small number of specimens, n &lt; 50. To evaluate differences in bond strength across the experimental groups, a two-way analysis of variance (ANOVA) was employed, and multiple comparison Tukey's post-hoc tests were used. Additionally, failure modes were analyzed using chi-square tests to determine significant associations between categorical variables. The tests were performed at a confidence level of 95% and with a p-value of &lt; 0.05 to represent statistical significance.

## Results

The Shapiro-Wilk test for normality applied to mean shear stress values indicated that all data groups were normally distributed (P&gt;0.05). Additionally, the Levene's test for homogeneity of variances confirmed that the assumption of equal variances across groups was met (P&gt;0.05). Two-way ANOVA revealed significant effects for tooth type, bonding material, and interaction (P&lt;0.05), Table 2.

Table 2

Across all the bonding materials tested, 3D-printed artificial teeth consistently exhibited lower shear bond stress than milled teeth. The descriptive statistics (mean and standard deviation) for the bond strength values are presented in Table 3.

Table 3

**Figure 3:**
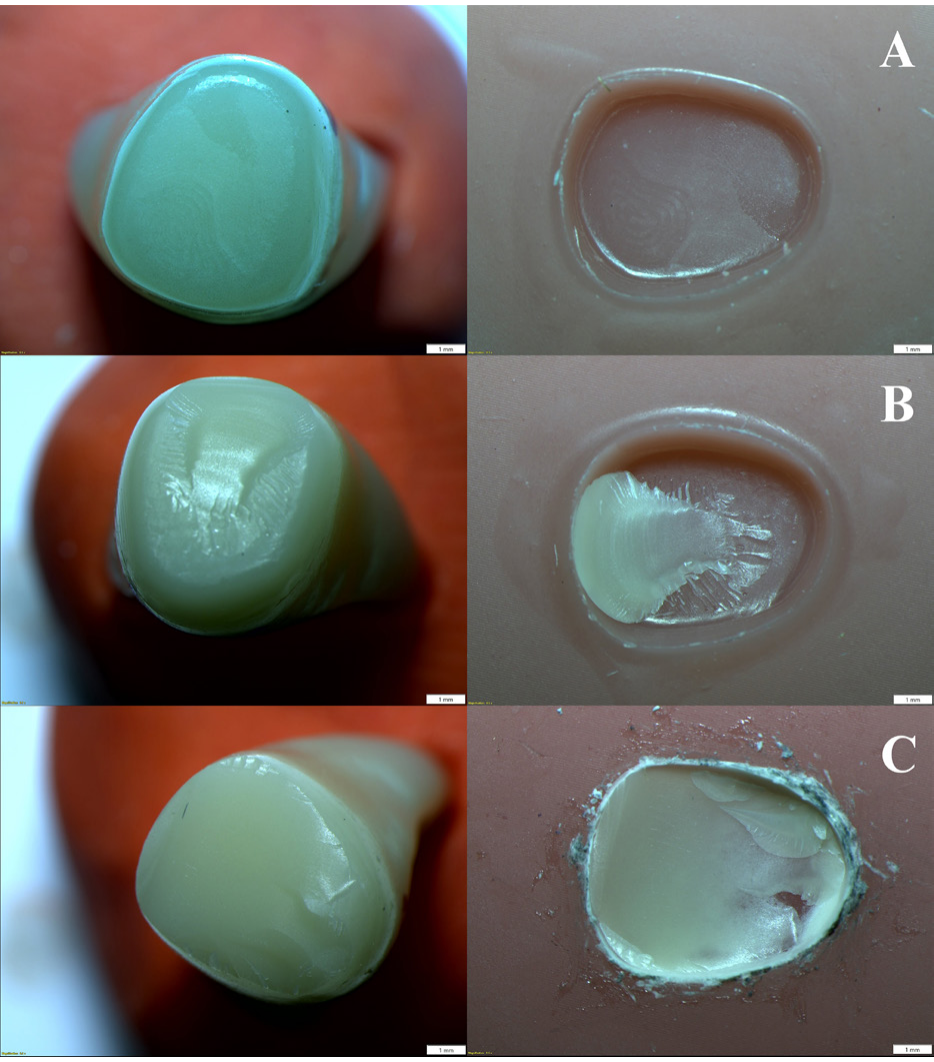
Mode of failure of fractured specimens. (A) Adhesive failure, (B) Mixed failure, and (C) Cohesive failure.

**Figure 4:**
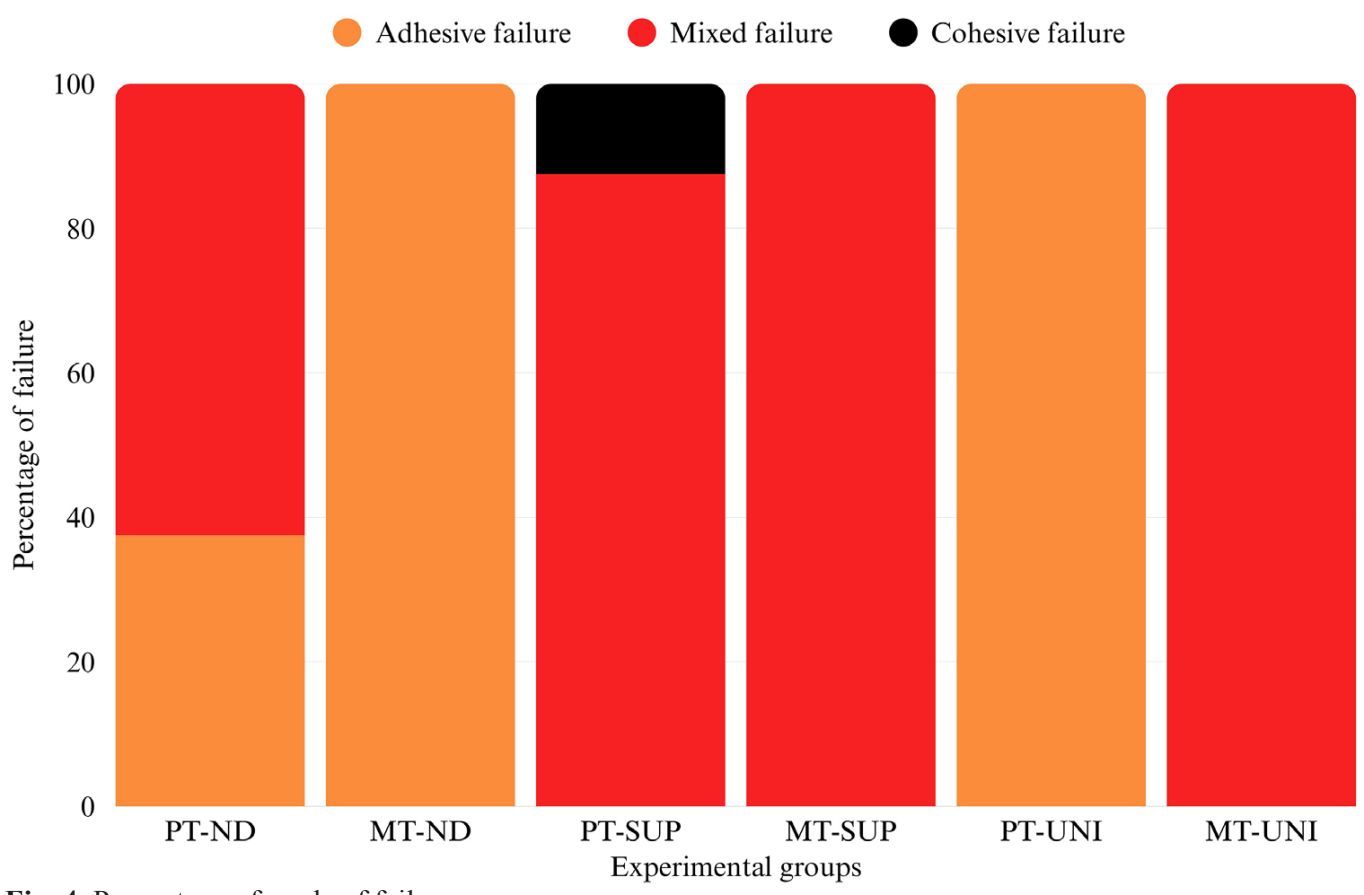
Percentage of mode of failure.

The highest mean shear stress value was observed in the MT-UNI, and the lowest value was observed for the MT-ND group. For 3D-printed teeth (Groups 1, 3, 5), 3D printed teeth bonded with Super-bond (Group 3 = 45.59 ± 3.47 N; [PT-SUP]) exhibited significantly higher bond strength than those bonded with NextDent (Group 1 = 34.12 ± 3.38 N; [PT-ND] as a control group) and Unifast (Group 5 = 23.68 ± 4.14 N; [PT-UNI]), respectively. In contrast, milled teeth (Groups 2, 4, 6) exhibited substantially higher bond strength with Super-bond (Group 4 = 87.34 ± 2.41 N; [MT-SUP]) and Unifast (Group 6 = 92.33 ± 4.90 N; [MT-UNI]), but markedly lower bond strength with NextDent (Group 2 = 11.72 ± 1.94 N; [MT-ND]). No statistically significant difference was found between the shear stress of the MT-UNI and MT-SUP groups. A different mode of failure was observed among the specimen groups. A mixed failure was most prevalent in the PT-ND and PT-SUP groups. All specimens in the MT-ND and PT-UNI groups exhibited 100% adhesive failure, while those in the MT-SUP and MT-UNI groups displayed 100% mixed failure, as represented by percentage (Figs. 3,4).

3

4

## Discussion

This study demonstrated that both denture tooth manufacturing methods and bonding materials significantly affect the shear bond strength to 3D-printed denture bases, thereby rejecting the null hypothesis that these factors had no influence. In the control group (PT-ND), specimens were fabricated using standard laboratory protocols. The printed tooth was bonded with the denture base using a small amount of the same denture base resin applied at the bonding interface. After assembly, all specimens underwent a post-curing process according to the manufacturer's recommendations to ensure complete polymerization. Milled teeth bonded to 3D-printed denture bases were selected for this study because they provide superior esthetics and better wear resistance. This choice is critical because excessive wear reduces occlusal vertical dimension, impairing masticatory function and compromising facial aesthetics over time. It also offers favorable cost efficiency compared to monolithic, milled CAD/CAM dentures. Previous studies have described various techniques for bonding prefabricated teeth to 3D-printed denture bases, such as light-polymerized adhesives and heat polymerization methods (14 - 16). However, these approaches often require specialized equipment, extended processing time, and laboratory involvement, necessitating denture removal from the patient. Super-bond and Unifast Trad were selected for this study as they are widely available in dental clinics and require no additional equipment or laboratory procedures. Their selection was based on optimizing clinical workflow while maintaining practical application. The experimental design compared bond strength and durability while standardizing printing parameters. Anterior teeth were tested per modified ISO 19736 guidelines, with a C-clamp ensuring consistent positioning to avoid skewed results (17). This study highlights key differences in bonding performance between milled and printed teeth. Milled PMMA teeth exhibit comparable bond strength with both Super-bond® and Unifast Trad. These are plausibly explained by their dense, uniform structure and chemical compatibility with methyl methacrylate (MMA) based adhesive. The 4-META monomer in Super-bond enhances polymerization, while Unifast Trad's MMA solvent action on PMMA surfaces improves bonding through polymer swelling, consistent with findings by Vallitty and Ryter (18). The extent of swelling depends on the polymer's cross-linking density. PMMA disks are produced under conditions of intense heat and pressure in an entirely dry setting, forming a densely crosslinked polymer-monomer network. In recent years, multiple companies have introduced different versions of PMMA disks to the market. However, specific technical details about their composition and production remain confidential due to intellectual property protections. Contrary to the findings of Takahashi et al. and Suzuki et al., which suggested that higher cross-linking in teeth reduces bond strength with denture base resins (19 , 20), our study observed no such correlation. Additionally, some research has found no significant relationship between the chemical composition or cross-linking of PMMA in denture teeth and their bond strength to denture base resins (9). These discrepancies may arise from variations in experimental methodologies. In addition, many studies confirm that MMA-containing materials improve CAD/CAM PMMA bonding, with performance comparable to heat polymerization (21 - 23). In contrast, printed teeth show weaker adhesion, especially with Unifast Trad, due to their layered 3D-printed structure, incomplete polymerization, and residual monomers that hinder bonding penetration. These structural limitations create interfacial weaknesses and micro-gaps, resulting in inferior bonding compared to milled teeth (24 , 25). The study used NextDent C&amp;B MFH, composed primarily of ethoxylated bisphenol A dimethacrylate (EBPADMA). Even after curing, printed teeth retain unpolymerized dimethacrylate, allowing Super-bond's MMA resin and 4-META monomer to form stronger chemical bonds. This explains Super-bond's significantly higher shear bond strength compared to Unifast Trad's simpler PMMA/MMA composition. Super-bond's superior performance stems from its 4-META adhesive, MMA matrix, and TBB catalyst. This catalyst allows the glue to harden at room temperature without shrinking or warping. Unlike other materials, Super-bond does not just stick to the surface; it forms an interpenetrating polymer network (IPN) between the existing PMMA matrix (in artificial teeth or denture base) and the newly polymerized resin, creating a tight mechanical grip similar to Velcro. This results in a mechanically interlocked network that resists debonding. Additionally, because it fully cures with minimal leftover chemicals, it's both strong and safe for dental use. Bonding performance is assessed by failure mode: adhesive (least desirable), mixed (acceptable), and cohesive (ideal) failures (26). The failure mode analysis further corroborated the quantitative findings. High bond strength groups (MT-SUP, MT-UNI, PT-SUP) predominantly exhibited mixed failure, indicating robust interfacial bonding. In contrast, low-strength groups (PT-UNI, MT-ND) showed adhesive failures, reflecting weaker interfacial adhesion. This trend supports the interpretation that bonding strength is both material- and substrate-dependent. Super-bond demonstrated reliable performance across fabrication methods, with 87.5% mixed failures on printed teeth and 100% mixed failures on milled teeth. A single cohesive failure in PT-SUP suggested that printed tooth material may be the limiting factor. In contrast, Unifast Trad had 100% adhesive failures with printed teeth but performed well with milled teeth (100% mixed failures), indicating substrate sensitivity. NextDent showed only adhesive failures, highlighting the need for surface treatments. Although mixed failures signify superior bond strength, they complicate repairs by preventing repositioning. Clinically, Super-bond is a dependable choice for both milled and printed teeth, whereas Unifast Trad and NextDent may require additional surface preparation for optimal results with printed substrates. Comparisons with previous studies were difficult because of the different experimental designs, material combinations, and printer technologies used (10 , 12 , 27). Previous studies have indicated that 3D-printed bases and 3D-printed teeth exhibit lower bond strength than conventional PMMA-based prostheses (27). The present study found that replacing bonding material with Super-bond significantly improved adhesion for both 3D-printed and milled teeth compared to the control (p &lt; 0.05). However, the bond strength values of the MT-ND group showed the lowest bond strength, and all specimens showed adhesive failure. Therefore, milled PMMA teeth are not optimal for combining with a printed resin. These results are in agreement with a study of Aydin(10) that showed the lowest bond strength in the conventional PMMA teeth and printed base (CT-PB) group. From the results of our study, we can improve the bond strength of milled teeth bonded to a 3D-printed base by using Unifast or Super-bond, yielding significantly higher bond strength values than the control group (p &lt; 0.05). While these in vitro results are promising, these findings should be interpreted with certain limitations in mind. The laboratory conditions used in this study cannot entirely simulate the dynamic oral cavity, which involves temperature changes, pH fluctuations, and masticatory forces. Moreover, it is not possible to evaluate the use of dentures in the long term. Another limitation could be testing only three bonding materials and two fabrication methods, which represent a small subset of available options in prosthetic dentistry, thus limiting the generalizability of the conclusions. Force units were also reported in Newtons rather than MPa may hinder comparability. Future studies should be carried out to enhance prosthetic longevity.

## Conclusions

Within the limitations of the present study, it was possible to conclude that this study provides evidence that careful selection of both artificial tooth material and bonding agent can significantly enhance the mechanical integrity of 3D-printed dentures. The study found that milled teeth bond more strongly to 3D-printed denture bases than printed teeth. For high-load bearing regions or complete dentures where long-term retention is critical, milled teeth combined with either auto-polymerizing resin (Unifast Trad) or Super-bond offer optimal performance, while only MMA-based resin cement with 4-META (Super-bond®) performs best with printed teeth. These findings support the integration of Super-bond into chairside and laboratory workflows, especially when working with emerging digital materials. Enhanced bonding not only increases prosthesis longevity but also reduces repair frequency and chair time, improving both patient satisfaction and clinical efficiency.

## Figures and Tables

**Table 1:** Materials used for the union of the denture tooth to the denture base.

Material	Manufacturer	Lot/Bach	Type of Material	Application
NextDent Denture 3D+	Vertex-Dental B.V.	WU313N03	3D-printed resin	Denture base
NextDent C&B MFH	Vertex-Dental B.V.	WT162N03	3D-printed resin	Teeth
Multi PMMA disk	Upcera	HI240513	Pre-polymerized PMMA	Teeth
Super-Bond	Sun Medical	FK2124	Self-cured resin cement	Bonding material
UNIFAST TRAD	GC Corporation	Powder: 2404163Liquid: 2404041	Auto-polymerized PMMA	Bonding material

1

**Table 2:** Two-way ANOVA of the influence of tooth type and bonding materials, and their interactions on the shear stress.

Source of variation	Sum of squares	df	Mean square	F	p-value
Intercept	115856.401	1	115856.401	9364.608	<0.001*
Tooth type	10325.333	1	10325.333	834.591	<0.001*
Bonding materials	17061.905	2	8530.953	689.552	<0.001*
Tooth type * Bonding materials (Interaction)	17506.850	2	8753.425	707.534	<0.001*
Error	519.613	42	12.372		
Total	161270.103	48			

*Significant at p-value <0.05

**Table 3:** Mean shear bond strength (N).

Group	Shear bond strength (SD)	Min.	Max.
PT-ND	34.12 ± 3.38 a	30.00	40.53
MT-ND	11.72 ± 1.94 b	9.65	14.73
PT-SUP	45.59 ± 3.47 c	40.40	49.45
MT-SUP	87.34 ± 2.41 d	83.23	90.83
PT-UNI	23.68 ± 4.14 e	15.53	28.25
MT-UNI	92.33 ± 4.90 d	85.75	98.68

*The same superscript letters show no statistically significant difference (p>0.05)

## Data Availability

The datasets used and/or analyzed during the current study are available from the corresponding author.
